# Supplementary material for: Plastic pollution in riverbeds fundamentally affects natural sand transport processes
Source: Commun Earth Environ. 2023 Jul 14;4(1):255. doi: 10.1038/s43247-023-00820-7 (PMC11041772; doi:10.1038/s43247-023-00820-7)
Supplement: Supplementary file 3 — Description of Additional Supplementary Files [file 43247_2023_820_MOESM3_ESM.pdf]

Description of Additional Supplementary Files

Below is a description of Supplementary Movie 1.

Clip 1 (00:00:00-00:00:08)

Flow direction is from right to left

Description: Migration of uncontaminated dunes. Sand is eroded from the stoss side and redeposited on the lee side of the dune, such that it steadily migrates downstream.

Associated Figures: Fig. 1a

Clip 2 (00:00:08-00:00:27)

Flow direction is from left to right

Description: 12 mm Mardi Gras bead sits on the lee slope of the migrating dune in incipient motion until it is stabilized and covered by sand and buried as an individual particle.

Associated Figures: Figs 1bii; 1biii

Clip 3 (00:00:27-00:00:37)

Flow direction is from right to left

Description: 14 mm Mardi Gras bead at the bottom of a lee slope rolls around in the recirculation zone prior to incorporation and burial at the base of the lee slope.

Associated Figures: Fig. 1bii

Clip 4 (00:00:37-00:00:59)

Flow direction is from right to left

Description: A group of low mobility plastic particles cluster, deflecting the flow and allowing higher mobility particles to aggregate and become buried.

Associated Figures: Fig. 1biii

Clip 5 (00:00:59-00:01:16)

Flow direction is from right to left

Description: Plastic particles with high mobility accumulate to a sufficient level to undertake multi-layered deposition.

Associated Figures: Figs 1bi; **2a**

Clip 6 (00:01:16-00:05:29)

Flow direction is from right to left

Description: A recently initiated dune begins to accumulate plastic on its lee slope, which then becomes incorporated into the dune as it advances. Notably, two cigarette filters are incorporated and later eroded (at 00:03:57 and 00:04:42), causing the dune to adjust, reduce its volume, and wash out. The pit formations induce the formation of small bedforms that migrate up the stoss slope to the crest. Note at 00:3:42, a string is formed by an 8 mm bead that has a 4 mm bead stuck in it; the combined properties of the beads caused it to behave differently with (a higher RD value) and promote the deposition of high mobility plastic particles.

Associated Figures: Figs 1b; 1c; 1d; **2c**

Clip 7 (00:05:29-00:06:34)

Flow direction is from right to left

Description: *Clips 7 and 8 are from the same dune*. A group of plastic particles is eroded from the stoss side of the dune causing an increased rate of erosion and slope steepening.

Associated Figures: Figs 1ci; 1d; **2b**

Clip 8 (00:06:34-00:07:49)

Flow direction is from right to left

Description: *Clips 7 and 8 are from the same dune*. From the beginning of the clip, the slope steepening caused by erosion of the group of plastic particles in Clip 7, is seen to migrate towards the dune crest. The resulting dune shape is markedly more symmetrical and lower in volume.

Associated Figures: Figs 1ci; 1d; **2b**

Clip 9 (00:07:49-00:08:28)

Flow direction is from right to left

Description: Changes to dunes upstream secondarily impacts this dune as it adjusts its shape to re-equilibrate to the flow conditions via a symmetrical dune form.

Associated Figures: Fig. 1d

Clip 10 (00:08:28-00:10:00)

Flow direction is from right to left

Description: Plastic particles become exposed on the stoss side of a migrating dune and erosion is enhanced around the obstacle. Whilst the particles themselves are not eroded out of the bed, the comparative scales of the obstacle and dune see that the dune becomes washed out.

Associated Figures: Figs 1ci; 1d
